# Long-term outcome of COVID-19 patients with acute kidney injury requiring kidney replacement therapy

**DOI:** 10.1186/s44158-024-00163-5

**Published:** 2024-05-09

**Authors:** Ilaria Godi, Laura Pasin, Andrea Ballin, Gabriele Martelli, Claudio Bonanno, Francesco Terranova, Enrico Tamburini, Caterina Simoni, Ginevra Randon, Nicola Franchetti, Leda Cattarin, Federico Nalesso, Lorenzo Calò, Ivo Tiberio

**Affiliations:** 1 Department of Urgency and Emergency, Anaesthesiology and Intensive Care Unit, University Hospital of Padua, Via Giustiniani 2, Padua, Italy; 2 Department of Medicine, Section of Anesthesiology and Critical Care, University of Padua, Padua, Italy; 3 Department of Nephrology and Dialysis, University Hospital of Padua, Padua, Italy

**Keywords:** COVID-19, Acute kidney injury, Kidney replacement therapy, Mortality, Kidney recovery

## Abstract

**Background:**

Limited data existed on the burden of coronavirus disease 2019 (COVID-19) renal complications and the outcomes of the most critical patients who required kidney replacement therapy (KRT) during intensive care unit (ICU) stay. We aimed to describe mortality and renal function at 90 days in patients admitted for COVID-19 and KRT.

**Methods:**

A retrospective cohort study of critically ill patients admitted for COVID-19 and requiring KRT from March 2020 to January 2022 was conducted in an Italian ICU from a tertiary care hospital. Primary outcome was mortality at 90 days and secondary outcome was kidney function at 90 days.

**Results:**

A cohort of 45 patients was analyzed. Mortality was 60% during ICU stay and increased from 64% at the time of hospital discharge to 71% at 90 days. Among 90-day survivors, 31% required dialysis, 38% recovered incompletely, and 31% completely recovered renal function. The probability of being alive and dialysis-free at 3 months was 22%.

**Conclusions:**

Critically ill patients with COVID-19 disease requiring KRT during ICU stay had elevated mortality rate at 90 days, with low probability of being alive and dialysis-free at 3 months. However, a non-negligible number of patients completely recovered renal function.

## Introduction

The morbidity and mortality associated with severe illness from coronavirus disease 2019 (COVID-19) is attributed to multiorgan injury, including kidneys [1, 2]. Patients experiencing severe acute kidney injury (AKI) due to COVID-19 disease may require kidney replacement therapy (KRT).

AKI-KRT is one of the most severe complications and demands a significant allocation of resources. During COVID-19 pandemic where staff and material resources were scarce, clinicians faced the difficulty to decide to treat COVID-19-associated AKI with KRT, without knowing the long-term impact of this decision. The high morbidity and mortality showed in severely ill non-COVID19 patients may suggest a similar outcome, but data are limited.

Since the first COVID-19 victim was recorded in Europe in the North East of Italy, SARS-CoV-2 has become a relentless epidemic in Italy, and the surge of critically ill patients needing intensive care resulted in an unexpected crisis raised important questions about clinical course and prognosis for these patients. Efforts were made to prevent the breakdown of the healthcare system, by adopting local measures, increasing ICU and medical ward capacity, and sharing therapeutic strategies [3]. At that time, the impact of COVID-19 disease on the kidneys was undefined and unclear, and the taskforce did not consider the potential need for KRT machine and expert staff. It is now critical to understand the burden of COVID-19 renal complications and the outcomes of patients treated with KRT for resource planning and to support clinicians, patients, and their families in the process of shared decision.

We describe the clinical characteristics and outcomes of mortality and kidney recovery of critically ill patients with COVID-19 disease who developed severe AKI requiring KRT from a tertiary care hospital severely hit during the pandemic. We also aimed to estimate the probability of being alive and dialysis-free at 3 months since KRT initiation.

## Material and methods

### Population

All consecutive adult patients requiring KRT after being admitted to the general ICU of Padua from March 2020 to January 2022 with laboratory-confirmed COVID-19 disease were included in the retrospective study.

Exclusion criteria were as follows: (1) history of end-stage kidney disease; (2) reason for ICU admission not related with COVID-19 disease (example: acetaminophen intoxication in a patient that tested positive for COVID-19).

Indications for KRT, as well as the modality chosen, were determined by consensus between the attending intensivists and nephrologists and based on clinical status of the patient. Specifically, in the case of moderate/severe AKI, the indication was considered for patients who showed low tolerance to volume overload or metabolic acidosis; in the case of medically refractory hyperkalemia, metabolic acidosis, or severe hypoxia due to volume overload, the indication was mandatory.

### Study outcomes

The primary outcome measure of the study was mortality at 90 days after initiation of KRT. The secondary outcome was kidney function at 90 days, measured as kidney recovery and dialysis dependence. Moreover, the probability to be alive and free of dialysis at 90 days was analyzed.

### Definitions

The diagnosis of COVID-19 was defined as a positive RT-PCR (real-time polymerase chain reaction) result.

AKI diagnosis and severity were based on Kidney Disease Improving Global Outcomes (KDIGO) criteria [4]. Baseline serum creatinine (SCr) was defined as the lowest value 365–7 days prior to hospitalization. If a prehospital baseline SCr was not available, we used SCr value obtained from Chronic Kidney Disease Epidemiology Collaboration estimation formula. Renal recovery was defined according to the Acute Disease Quality Initiative (ADQI) consensus as full recovery is the absence of AKI criteria; partial recovery can then be defined as a fall in AKI stage [5].

### Data collection

We collected data by detailed chart review and used a standardized patient report form to enter data into a secure online database (REDCap). Patient-level data included demographics, such as age, sex, body mass index (BMI), and comorbidities, such as chronic kidney disease (CKD), hepatic failure, cardiac failure, chronic obstructive pulmonary disease (COPD), diabetes mellitus (DM), antecedent solid neoplasm, active solid neoplasm, active hematological neoplasm, arterial hypertension, vasculopathy, and immunosuppression. We also collected the baseline SCr. Indication for KRT was recorded (fluid overload, uremia, hyperkalemia, acid–base disorders).

Characteristics at initiation of KRT were collected: heart rate, presence of arrhythmia, temperature, use of vasoactive, vasoactive inotropic score (VIS), mean arterial pressure (MAP), arterial partial pressure of oxygen over fractional inspiratory oxygen ratio (paO2/FiO2), arterial partial pressure of carbon dioxide (paCO2), pH, lactate, serum creatinine, urinary output (UO) during the day before start of KRT, urea, bilirubin, albumin, platelets count, fibrinogen, procalcitonin (PCT), C reactive protein (CRP), white blood cells count (WBC), need for mechanical ventilation, use of extracorporeal support, such as extracorporeal membrane oxygenation (ECMO) or extracorporeal dioxide removal (ECCO2R). Scores of illness severity (Sequential Organ Failure Assessment, SOFA, Simplified Acute Physiology Score, SAPS2) were collected at the time of KRT initiation.

We recorded the time from ICU admission and KRT initiation, as well as initial KRT modality and prescription (net ultrafiltration, prescribed dose, use of regional citrate anticoagulation or systemic heparin coagulation). We recorded complications related to KRT (such as hypomagnesemia, hypophosphatemia, hemorrhage, citrate accumulation, thromboembolism).

Kidney laboratory data were recorded also at ICU discharge, hospital discharge, and at 90 days. Data on long-term follow-up were gathered from patients’ electronic medical records.

### Ethical approval

The study was approved with a waiver of informed consent by the institutional review board “Comitato Etico per la Sperimentazione Clinica della Provincia di Padova” with protocol number 70601 on 27/01/022.

### Statistical analysis

The analysis was performed using R studio version 4.1.2.

Descriptive statistics were calculated for patients’ demographics, comorbidities, laboratory values, and hospital course and are presented as median (interquartile range) for continuous values or counts (percentage) for categorical values.

We evaluated differences between survivors and non-survivors using Mann–Whitney *U* test and chi-square test, as appropriate.

We used the package “survival” to estimate the probability of KRT independence at 90 days, with death as competing risk. The package “survminer” was used to plot the time-to-event function analysis. Patients were censored at 90 days.

In order to evaluate the influence of other variables on ICU mortality and 90-day mortality, we also performed backward stepwise multivariate analysis, using age, BMI, VIS, PaO2/FiO2, SAPS2, SOFA as continuous variables, and history of CKD, DM, arterial hypertension, or use of ECMO as categorical variables.

### Data availability

The data associated with the paper are not publicly available but are available from the corresponding author on reasonable request.

## Results

### Patients’ characteristics at baseline

**Fig. 1:**
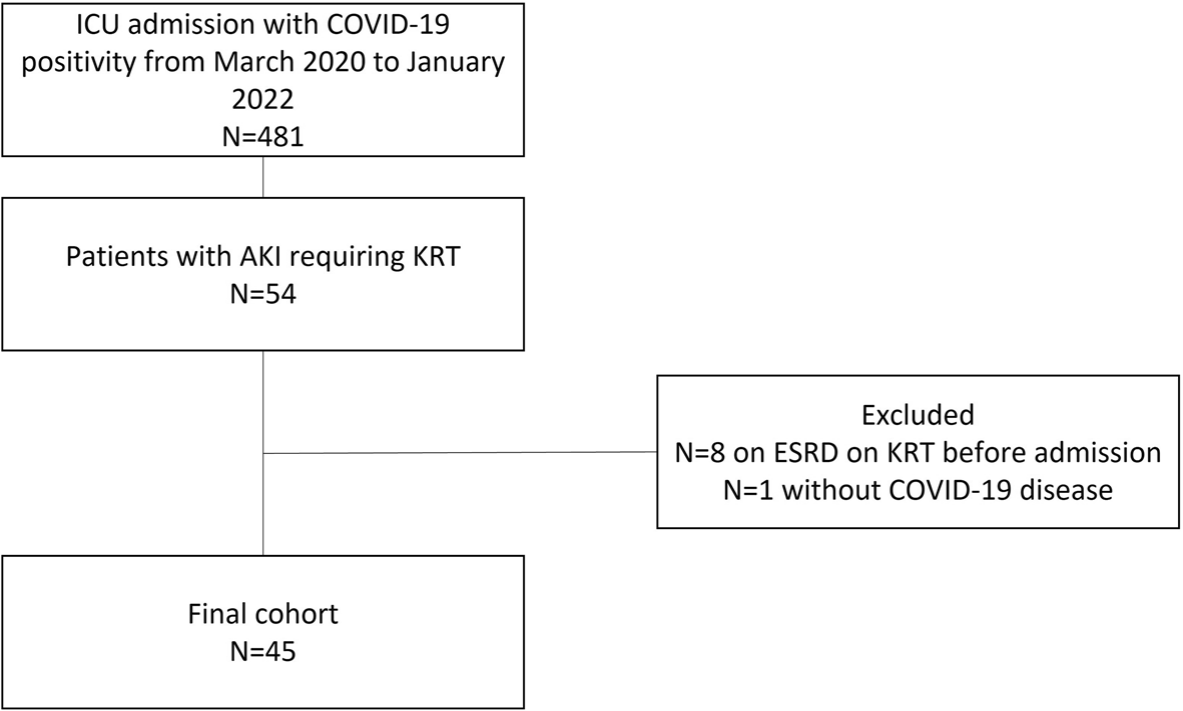
Study flowchart. ICU intensive care unit, COVID-19 coronavirus 2019, AKI acute kidney injury, KRT kidney replacement therapy, ESRD end-stage renal disease

During the study period, a total of 481 patients with COVID-19 positivity were admitted to ICU, among whom 54 patients (11.2%) were treated with KRT during ICU stay. We excluded eight patients that were on chronic dialysis before admission and one patient admitted to ICU for reasons unrelated to COVID-19 disease, as shown in Fig. 1. A final cohort of 45 patients was analyzed. The demographics of our cohort are outlined in Table 1. Basal creatinine was only present in 42% of our cohort and the median baseline sCr was 1 mg/dL (IQR 0.8–1.1); 27% had chronic kidney disease at any stage, with 22% with stages 3 and 4.

**Table 1:** Patients’ demographics and comorbidities. Differences between 90-day survivors and non-survivors are reported with *p*-value for significance

	Total cohort	90-day survivors	90-day non-survivors	*p*-value
Number of patients	45	13	32	
Demographics				
Age, years	69 (60–74)	69 (58–78)	68 (60–74)	0.854
Male sex, %	82	85	81	0.789
BMI, kg/m^2^	28 (26–33)	28 (26–30)	29 (26–33)	0.997
Comorbid conditions				
Baseline CKD, %	27	31	25	0.692
KDIGO stage 3 or more, %	22	23	22	0.584
Baseline SCr, mg/dl	1 (0.8–1.1)	1 (1–1.2)	1 (0.8–1.1)	0.854
Hepatic failure, %	2	0	3	0.519
Cardiac failure, %	2	0	3	0.519
COPD, %	2	0	3	0.519
DM, %	22	15	25	0.482
Antecedent solid neoplasm, %	9	15	6	0.329
Active solid neoplasm, %	4	8	3	0.5
Active hematological neoplasm, %	2	0	3	0.519
Arterial hypertension, %	67	77	62	0.352
Vasculopathy, %	18	23	16	0.553
Immunosuppression, %	9	8	9	0.857
Characteristics at ICU admission				
Organ failure > 2, %	22	25	15	0.482

*BMI* Body mass index, *CKD* Chronic kidney disease, *KDIGO* Kidney disease: improving global outcomes, *SCr* Serum creatinine, *COPD* Chronic obstructive pulmonary disease, *DM* Diabetes mellitus, *ICU* Intensive care unit, *KRT* Kidney replacement therapy

### Primary outcome

ICU mortality was 60% with a median LOS of 29 [17–38] days. Mortality increased from 64% at the time of hospital discharge to 71% at 90 days. Ninety-day non-survivors had significantly lower paO2/FiO2 at the time of KRT initiation and experienced more complications related to KRT during ICU stay (Table 2). The major risk factor for ICU mortality was the.history of CKD, as shown in the multivariate analysis in Table 3, while no specific risk factor was found for 90-day mortality (Table 4).

**Table 2:** Patients’ characteristics at initiation of KRT, KRT prescription, and KRT complications. Differences between 90-day survivors and non-survivors are reported with *p*-value for significance

	Total cohort	90-day survivors	90-day non-survivors	*p*-value
Number of patients	45	13	32	
Characteristics at initiation of KRT				
SOFA score	16 (14–18)	16 (13–18)	16 (14–18)	0.924
SAPS2	70 (62–75)	63 (54–75)	70 (64–75)	0.709
Mechanical ventilation, %	100	100	100	1
Vasoactive use, %	84	85	84	0.793
Extracorporeal support, %	9	0	12	0.182
ECMO, %	7	0	9	0.253
ECCO2R, %	2	0	3	0.519
Cardiac frequency, bpm	95 (83–106)	86 (64–101)	96 (85–107)	0.186
Arrhythmya, %	24	23	25	0.892
MAP, mmHg	79 (69–88)	80 (71–85)	79 (66–91)	0.924
Temperature, °C	36.5 (35.9–37.0)	36 (35.4–37.2)	36.6 (36–37)	0.573
VIS	23 (6–35)	19 (7–29)	25 (6–36)	0.573
paO2/FiO2, mmHg	115 (85–165)	151 (112–199)	107 (75–151)	0.024*
paCO2, mmHg	42 (39–47)	42 (39–44)	42 (40–54)	0.924
Ph	7.38 (7.32–7.42)	7.38 (7.34–7.43)	7.36 (7.31–7.42)	0.806
Renal characteristics				
Urine output, ml	1990 (1210–2900)	1800 (1390–2500)	2300 (1055–3100)	
Fluid overload, %	73	69	75	0.692
Uremia, %	56	46	59	0.419
Hyperkalemia, %	13	8	16	0.478
Acid–base disorder, %	9	8	9	0.857
SCr at KRT initiation, mg/dl	2.5 (1.9–4.1)	3 (2.4–5.0)	2.4 (1.8–3.9)	0.451
ICU to KRT length of stay, days	7 (2–13)	6 (1–13)	8 (2–14)	0.451
Laboratory parameters at KRT initiation				
Lactate, umol/L	2 (1.4–2.6)	1.6 (1.3–2.5)	2 (1.5–2.7)	0.792
Urea, mg/L	145 (90–232)	142 (76–231)	148 (96–233)	1
Bilirubin, mg/L	9 (5–19.5)	11 (3–19)	9 (5–20)	0.845
Platelet cells count, 10^3^/µL	207 (138–307)	224 (139–290)	202 (120–314)	0.924
Procalcitonine, ng/ml	1.4 (0.5–3.6)	1.9 (0.4–3.9)	1.4 (0.6–3.5)	0.925
C reactive protein, mg/L	110 (35–190)	110 (35–154)	110 (31–272)	0.775
White blood cells count, 10^9^/µL	15 (9.6–19.8)	14.3 (9.7–17.9)	16 (9.3–20.3)	1
KRT first prescription				
Continuous modality, %	100	100	100	1
Net ultrafiltration, ml	100 (100–120)	100 (100–100)	100 (100–150)	0.257
Prescribed dose, ml/kg/h	30 (28–35)	29 (27–34)	30 (27–35)	0.944
Anticoagulation, %				
Regional citrate anticoagulation, %	69	69	69	0.912
No anticoagulation, %	11	15	9	0.525
Systemic heparin, %	9	0	12	0.345
Complications related to KRT, %	82	61	91	0.021*
Hypomagnesemia, %	64	38	75	0.020*
Hypophosphatemia, %	80	61	87	0.048*
Thromboembolism, %	4	0	6	0.356
Hemorrhage, %	16	8	19	0.354
Citrate accumulation, %	2	0	3	0.519

*KRT* Kidney replacement therapy, *SOFA* Sequential organ failure assessment, *SAPS2* Simplified acute physiology score, *ECMO* Extracorporeal membrane oxygenation, *ECCO2R* Extracorporeal carbon dioxide removal, *MAP* Mean arterial pressure, *VIS* Vasoactive inotropic score, *paO2/FiO2* Partial Pressure of arterial oxygen content/fraction of inspired oxygen, *paCO2* Partial pressure of carbon dioxide content

^*^p-value < 0.05

**Table 3:** Multivariate analysis for ICU mortality

Variables	Odds ratio	95% confidence interval	*p*-value
Age	0.94	0.86–1.02	0.14
BMI	1.03	0.88–1.21	0.69
CKD	6.95	1.04–46.5	0.045*
DM	0.75	0.11–5.03	0.76
Arterial hypertension	0.93	0.16–5.38	0.94
VIS	1.04	0.97–1.11	0.24
PaO2/FiO2	0.99	0.98–1.01	0.77
SAPS2	1.02	0.95–1.1	0.49
SOFA	0.77	0.56–1.07	0.12
ECMO	1.55	0.95–4.72	0.14

*BMI* Body mass index, *CKD* Chronic kidney disease, *DM* Diabetes mellitus, *SOFA* Sequential organ failure assessment, *SAPS2* Simplified acute physiology score, *ECMO* Extracorporeal membrane oxygenation, *VIS* Vasoactive inotropic score, *paO2/FiO2* Partial pressure of arterial oxygen content/fraction of inspired oxygen

^*^p-value < 0.05

**Table 4:** Multivariate analysis for 90-day mortality

Variables	Odds ratio	95% confidence interval	*p*-value
Age	0.96	0.88–1.05	0.47
BMI	1.01	0.85–1.22	0.85
CKD	1.26	0.18–8.66	0.81
DM	0.52	0.07–4.1	0.53
Arterial hypertension	1.62	0.25–10.6	0.61
VIS	1.03	0.96–1.1	0.41
PaO2/FiO2	0.99	0.98–1.01	0.32
SAPS2	1.08	0.98–1.19	0.102
SOFA	0.81	0.58–1.13	0.21
ECMO	1.17	0.91–2.98	0.28

*BMI* Body mass index, *CKD* Chronic kidney disease, *DM* Diabetes mellitus, *SOFA* Sequential organ failure assessment, *SAPS2* Simplified acute physiology score, *ECMO* Extracorporeal membrane oxygenation, *VIS* Vasoactive inotropic score, *paO2/FiO2* Partial pressure of arterial oxygen content/fraction of inspired oxygen

### Secondary outcomes

**Fig. 2:**
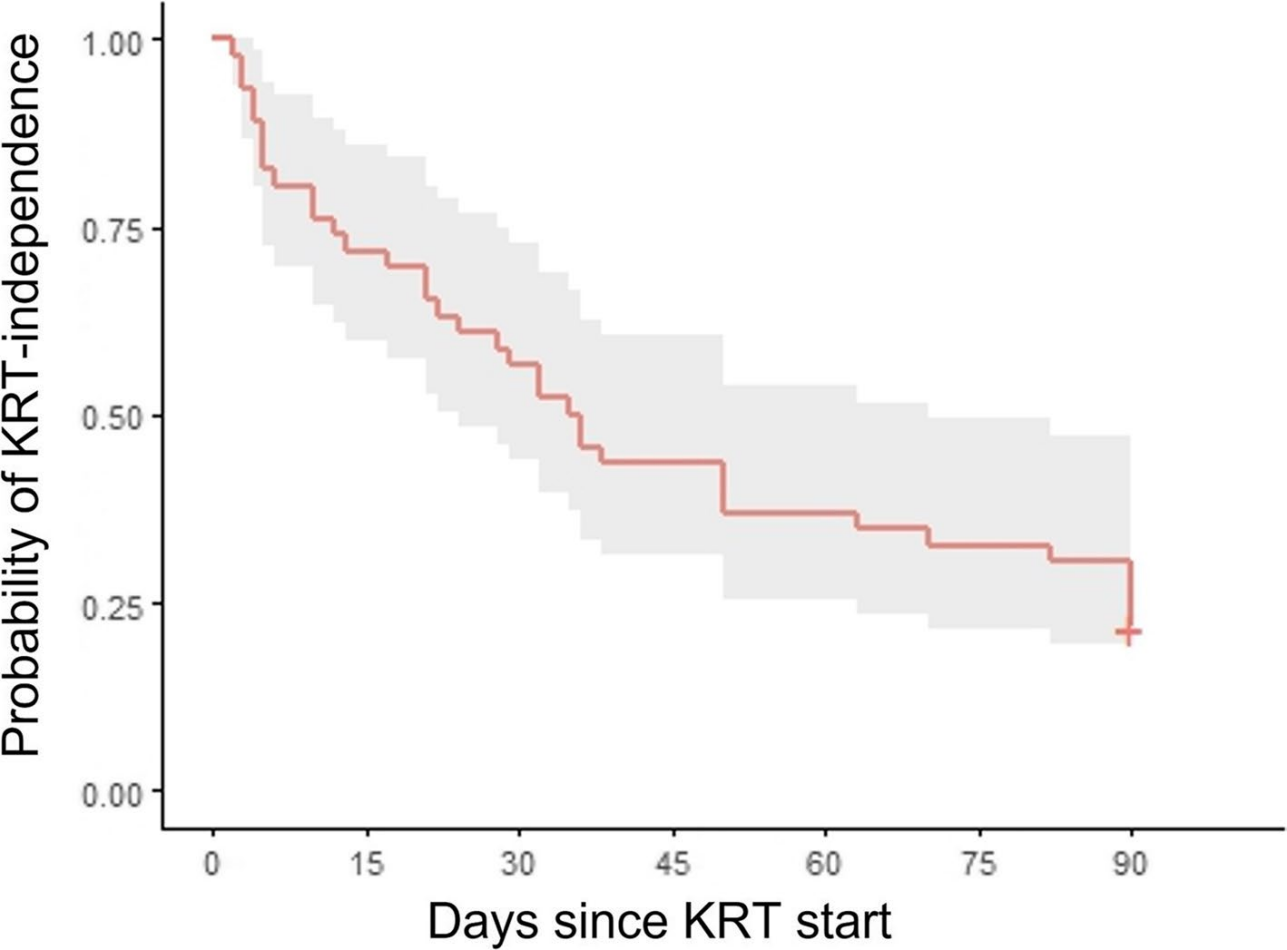
Probability of KRT independence at 90 days since KRT initiation. Time-to-event analysis with death as a competing risk

Among ICU survivors, the median ICU LOS was 32 [21–45] days, with a median time from KRT initiation to liberation of 20 [6–43] days. Among 90-day survivors, 4 patients (31%) still required dialysis, 5 patients (38%) recovered incompletely with a median eGFR of 42 [32–49] ml/min per 1.73 m^2^, and 4 patients (31%) completely recovered renal function with a median eGFR of 75 [68–77] ml/min per 1.73 m^2^. As shown in Fig. 2, the probability to be alive and free of dialysis at 90 days for a patient who developed AKI-KRT during the ICU stay for COVID-19 disease was 22%.

## Discussion

In this monocenter cohort study of 481 critically ill patients with COVID-19, we found that almost 10% of all patients required KRT for AKI over the disease course. During ICU stay, 60% of AKI-KRT patients died and we found history of CKD as only risk factor. The mortality rate of AKI-KRT patients rose to 71% at 90 days, while more than 20% of survivors still needed dialysis at 3 months. According to our estimation, the probability of being alive and dialysis-free at 3 months since KRT initiation was 22%. Cumulatively, these findings indicate that AKI-KRT is associated with high rates of mortality and morbidity. The present study is one of the few investigating mortality and renal recovery in the subpopulation of critically ill patients requiring KRT during the course of COVID-19 disease.

Several studies investigated long-term outcomes in critically ill patients with COVID-related AKI [2, 6–9], but few focused on the subpopulation of critically ill patients requiring KRT during COVID-19 disease.

During the first wave of pandemic, there were reports with a limited number of patients worldwide that showed different rates of mortality. In a small cohort of 13 Dutch patients, the ICU mortality rate was 39% [10], while a larger cohort of 114 patients in New York showed a 60-day mortality of 70% [11]. The reports did not specify many characteristics of the population analyzed particularly the severity and the comorbidities prior to ICU admission. Another small cohort of 34 Brazilian patients found a 60-day mortality of 35.4% in critically ill patients all mechanically ventilated and with high rate of ECMO [12]. Stevens et al. analyzed again in New York a cohort of 115 patients, of which 99% mechanically ventilated and 84% needing vasopressors, with a SOFA score of 15 [13]. The authors found a 51% mortality at follow-up. They identified COPD and coronary artery disease as main risk factors for mortality even though a small proportion of patients had history of these comorbidities. Another study by Eriksson et al. on 82 patients (SAPS2 of 61, all mechanically ventilated, with 8.5% use of ECMO) in Sweden demonstrated a rate of ICU and 30-day mortality of 39% and 45%, respectively [14]. The larger cohort represented by 876 AKI-KRT patients with COVID-19 from 67 ICUs in the USA was investigated in the STOP-COVID study by Gupta et al., which showed a 28-day mortality rate of 67% [15]. The analyzed cohort required mechanical ventilation in 98.1% of patients and 41% of the population had pre-existing kidney chronic dysfunction. Other more recent investigations reported a hospital mortality rate of 67.5%, 72.5%, 68.1%, and 43.4% in cohorts in Boston [16], Brazil [17], India, and Pakistan [18], respectively.

As discussed by Gupta et al., the different rates of mortality might be due to many patient-level (history of pre-existing CKD or high-risk population like Black and Hispanic races or severity of COVID-19 disease) and hospital-level factors (resource limit or high burden hospital) [15].

The population that we described was very sick, as expressed by severity scores, such as SAPS 2 and SOFA scores of 70 and 16, respectively. The entire cohort of 45 patients needed invasive mechanical ventilation and 84% required vasopressor use during ICU stay. Moreover, the pre-existing CKD was an independent risk factor for ICU mortality, while we could not find any independent factor associated with 90-day mortality. Pre-existing CKD has been among the most robust predictors of severe and critical illness in patients with COVID-19. A meta-analysis with data from 9 studies found that CKD was associated with a threefold higher risk of mortality in COVID-19 hospitalized patients [19]. Consistent with our finding, Eriksson et al. found that the baseline serum creatinine was a predictor for survival in critically ill patients with AKI-KRT [14]. Other predictors of mortality in COVID-19 critical patients needing KRT were age [14] and a higher number of organ dysfunction [17].

Renal recovery after AKI-KRT is important for patients, families, and all clinicians involved. Our study shows 9% (31% among survivors) of dialysis dependence at 90 days, while 9% (31% among survivors) completely recovered renal function according to sCr at follow-up.

Melero et al. investigated a subpopulation of critically ill COVID-19 patients who required KRT and reported a zero rate of dialysis dependence, but a high rate of renal non-recovery at 1 year [20]; in contrast with our analysis, only patients with normal baseline serum creatinine were analyzed, while in our cohort, 27% had history of CKD at any stage. In fact, Ng et al. published the outcomes among patients hospitalized with COVID-19 and AKI [21]. Among those with AKI-KRT who survived, 30.6% remained on dialysis at discharge, and pre-hospitalization CKD was the only independent risk factor associated with needing dialysis at discharge. Hsu et al. noticed that the more severe the pre-existing kidney dysfunction, the greater the risk for dialysis dependence in critically ill patients with AKI-KRT [22]. In another report from New York, a population of ICU patients with AKI-KRT, only 26% of survivors were able to be weaned from KRT before hospital discharge [11]. Chand et al. investigated specifically critically ill patients who developed AKI-KRT and survived; among 35 survivors, 77% were KRT liberated and 57% had complete renal recovery [23].

Comparing with other studies, we report a low rate of renal recovery, and there might be many possible explanations: the baseline comorbid state of our population (27% was known to have CKD at any stage), the severity of COVID-19 disease treated in our unit (where almost exclusively mechanically ventilated patients were admitted), the management of fluid therapy and drug stewardship, that were not taken into account in this analysis.

There are several potential limitations concerning the results of the present study. First, this was a single-center observational study which might impact the generalization of findings. Second, due to the retrospective nature of the study, some laboratory results were not available for all patients. We had incomplete information on treatment before ICU admission. Third, baseline sCr was available in less than half the patients: we evaluated renal recovery according to sCr value at follow-up and this might overestimate or underestimate the rate of recovery. Hence, further research might help shed light on the effect of COVID-19 on mortality and kidney disease.

## Conclusion

Critically ill patients with COVID-19 disease requiring KRT during ICU stay had elevated mortality rate at 90 days, with low probability of being alive and dialysis-free at 3 months. However, a non-negligible number of patients completely recovered renal function.

## Abbreviations

ADQI: Acute disease quality initiative
AKI: Acute kidney injury
BMI: Body mass index
CKD: Chronic kidney disease
COPD: Chronic obstructive pulmonary disease
COVID-19: Coronavirus disease 2019
CRP: C reactive protein
DM: Diabetes mellitus
ECCO2R: Extracorporeal dioxide removal
ECMO: Extracorporeal membrane oxygenation
eGFR: Estimated glomerular filtration rate
ICU: Intensive care unit
KDIGO: Kidney disease improving global outcomes
KRT: Kidney replacement therapy
MAP: Mean arterial pressure
paCO2: Arterial partial pressure of carbon dioxide
paO2/FiO2: Arterial partial pressure of oxygen over fractional inspiratory oxygen ratio
PCT: Procalcitonin
RT-PCR: Real-time polymerase chain reaction
SAPS2: Simplified acute physiology score 2
sCr: Serum creatinine
SOFA: Sequential organ failure assessment
UO: Urinary output
VIS: Vasoactive inotropic score
WBC: White blood count
